# The epidemiology of Varicella Zoster Virus infection in Italy

**DOI:** 10.1186/1471-2458-8-372

**Published:** 2008-10-27

**Authors:** Giovanni Gabutti, Maria C Rota, Marcello Guido, Antonella De Donno, Antonino Bella, Marta L Ciofi degli Atti, Pietro Crovari, the Seroepidemiology Group

**Affiliations:** 1 Dept. of Clinical and Experimental Medicine, Section of Hygiene and Occupational Health, University of Ferrara, Ferrara, Italy; 2 National Center of Epidemiology, Surveillance and Health Promotion, Istituto Superiore di Sanità, Rome, Italy; 3 Dept. of Biological and Environmental Sciences and Technologies, Lab of Hygiene, University of Salento, Lecce, Italy; 4 Dept of Health Sciences, Section of Hygiene and Preventive Medicine, University of Genoa, Genoa, Italy

## Abstract

**Background:**

The epidemiological importance of varicella and zoster and the availability of an efficacious and safe vaccine have led to an important international debate regarding the suitability of mass vaccination. The objective of the study was to describe the epidemiology of varicella and zoster in Italy and to determine whether there have been changes with respect to observations provided by an analogous study conducted 8 years ago, in order to define the most appropriate vaccination strategy.

**Methods:**

A number of data sources were evaluated, a cross-sectional population-based seroprevalence study was conducted on samples collected in 2004, and the results were compared with data obtained in 1996.

**Results:**

The data from active and passive surveillance systems confirm that varicella is a widespread infectious disease which mainly affects children. VZV seroprevalence did not substantially differ from that found in the previous study. The sero-epidemiological profile in Italy is different from that in other European countries. In particular, the percentage of susceptible adolescents is at least nearly twice as high as in other European countries and in the age group 20–39 yrs, approximately 9% of individuals are susceptible to VZV.

**Conclusion:**

The results of this study can contribute to evaluating the options for varicella vaccination. It is possible that in a few years, in all Italian Regions, there will exist the conditions necessary for implementing a mass vaccination campaign and that the large-scale availability of MMRV tetravalent vaccines will facilitate mass vaccination.

## Background

Varicella is a ubiquitous highly contagious infectious disease caused by the varicella zoster virus (VZV) [1]. After primary infection, VZV tipically remains latent in the dorsal root ganglia and after many years results in zoster [2]. Approximately 10–20% of adults in the course of their lifetime have an episode of zoster, which is usually characterized by unilateral radicular pain and a vescicular eruption generally limited to a dermatome. Of the zoster-related complications, the most common is post-herpetic neuralgia, which can considerably worsen an individual's quality of life [3]. The development of zoster seems to be associated with advanced age and decreased immune response (in particular the cell-mediated response) [4].

The epidemiological importance of varicella and zoster and the availability of an efficacious and safe vaccine [5,6] have led to an important international debate regarding the suitability of mass vaccination. At the European level, the European Working Group on Varicella (EuroVar) has recently recommended that routine varicella vaccination be performed for healthy children 12–18 months of age and for all susceptible children before 13 years of age, in addition to catch up for older children and adults who are anamnestically negative for the infection and at high risk of transmission, exposure, or complications. However, mass vaccination is recommended only for countries where high vaccination coverage can be rapidly achieved and maintained [7]. This is to avoid problems that have occurred in the past with measles, mumps, and rubella; in particular, the sub-optimal vaccination coverage has reduced, but not stopped, viral circulation, creating a pool of susceptible individuals among older children and adults [8].

In Italy, although varicella is subject to statutory notification, it is often underreported. Thus the data provided by routine notification need to be validated and integrated with data from other sources, such as sentinel surveillance systems or sero-epidemiological investigations. This is especially important when evaluating whether or not to perform mass vaccination or assessing the impact of vaccination campaigns that have already been implemented. The objective of the present study was to describe the epidemiology of varicella and zoster in Italy using a number of data sources and to determine whether there have been changes with respect to observations provided by an analogous study conducted 8 years ago, in order to define the most appropriate vaccination strategy.

## Methods

### Incidence data

In Italy, varicella is subject to mandatory notification, and all reported cases are recorded by Italy's National Census Bureau (ISTAT). For zoster, notification is not mandatory; thus no national-level data are available.

We analysed ISTAT data on cases of varicella for the period 1991–2004. We determined the trend in crude incidence per 100,000 inhabitants, using as reference the Italian population included in national censuses (for the years 1991 and 2001) or estimates provided by ISTAT (for the remaining years). Moreover, we calculated the trend in incidence for the periods 1991–1995, 1996–2000, and 2001–2004, by age class: 0–14, 15–24, 25–64, and ≥65 years. For each of these periods, the trend in incidence by geographic area was also evaluated (northern Italy, central Italy, and southern Italy and the islands).

### Evaluation of other databases

To conduct a more in-depth epidemiological evaluation, we analysed data from other databases with information on varicella and zoster. With specific regard to varicella, we considered the incidence data from Italy's Paediatric Sentinel Surveillance System of Vaccine-Preventable Diseases (SPES) for the years 2000–2005. Surveillance consists of collecting data from a network of paediatricians located throughout Italy. In the period considered, approximately 320–470 paediatricians participated, with a national coverage of approximately 2.5–5% for persons 0–14 years of age.

We also examined the National Hospital Discharge Database, created in 1994, which collects information on all hospitalizations recorded in Italy [9]. For the analysis of the data from this latter database, which is freely available from the web site of the national Ministry of Health, we considered the main reason for hospitalization, which is codified using ICD9-CM (code 052 for varicella and code 053 for zoster). The analysis was performed on data for the period 2000–2003.

Finally, we reviewed data on mortality due to varicella and zoster for the years 1991–2002, provided by ISTAT.

### Seroprevalence study

A national cross-sectional population-based seroprevalence study of varicella antibodies was conducted in compliance with the Helsinki Declaration and with the Law Decree n. 196/2003, article 24 (Code for the protection of personal data). In the period from January 2003 to October 2004, blood samples were collected from at least one reference laboratory in eighteen out of Italy's 20 Regions, so that the sample would be geographically representative. The samples had been taken for diagnostic purposes or routine ascertainment and had been frozen at -20°C until use and were analyzed by the same national reference laboratory (University of Salento, Lecce).

For each person from whom blood was collected, the purpose of the study was explained and oral informed consent for using the sample for the study was obtained. Specimens were collected anonymously and only age, sex and date of sampling were recorded. We excluded from the study immunocompromised patients, those who had received a blood transfusion in the previous 6 months, and those with an acute infectious disease. Each Regional reference laboratory was asked to collect 6 samples for each year of age in the 0–20-year age group, 10 samples for each 5-year range in the 21–40-year age group, and a total of 10 samples for the 41–50-year age group. The samples had to be equally representative of males and females. A total of 3,094 serum samples were collected.

Varicella antibodies were quantified using an immunoenzyme micro-method (Enzygnost anti-VZV-virus/IgG, Dade Behring GmbH), which has a high sensitivity and specificity (respectively, 99.3% and 100%).

The following criteria were applied for the qualitative evaluation:

- IgG negative sample ΔE < 0.100 (cut-off)

- IgG positive sample ΔE > 0.200

- Equivocal IgG sample 0.100≤ΔE≤0.200

The equivocal samples were retested: if the result was confirmed, the sample was classified as "equivocal". The IgG positive samples were quantitatively evaluated using the following formula: Log_10 _mIU/ml = α × ΔE^β^, where α and β represent lot-dependent constants. Antibody activity was expressed in mIU/ml, in accordance with the international standard for varicella-zoster immunoglobulin of the World Health Organization; the cut-off for the test was 50 mIU/ml, equivalent to a ΔE of 0.100.

### Statistical analysis

Direct standardized incidence rates were calculated by using national 1991 census data as the reference population.

Seroprevalence data were summarized as percentages with 95% confidence interval (95%CI) and positive antibody titres presented as geometric means. Differences among percentages of seropositive subjects were assessed by the chi-square test, while differences among geometric titres were assessed by Student's t test of logarithmically transformed values.

Data were also analysed by gender and geographical area, and were then compared with results obtained from a seroprevalence study conducted with the same test method and cut-off in 1996 [10].

A multiple logistic regression model was used to determine the relationship between antibody titres (positive versus negative) and a set of explanatory variables. The following variables were included in the model: sex, age group, geographical area and year of sample collection. The likelihood ratio test was used to compare different models. Statistical analysis was performed by Stata software version 9.2.

## Results

### Incidence data

**Figure 1:**
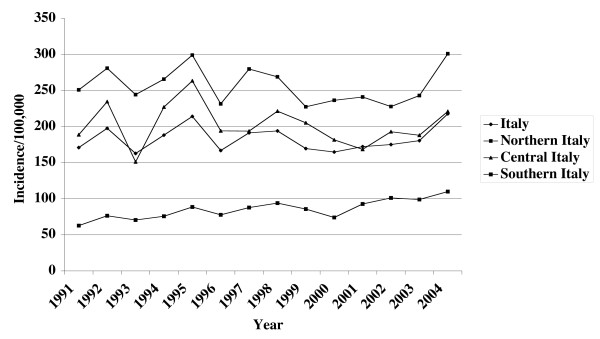
Varicella in Italy: area specific and national incidence rates/100,000 population, 1991–2004.

Based on the data provided by ISTAT, varicella was confirmed to be a disease which each year affects a large proportion of the population (Figure 1). The overall standardized annual incidence ranged from 164.4 to 244.2 per 100,000 population in the years 1991–2004. For all three periods considered (1991–1995, 1996–2000 and 2001–2004), it clearly emerged that varicella mainly affects children (0–14 years). Moreover, in this age group, the reported incidence significantly increased, from 996 per 100,000 population in 1991–1995 to 1,164 in 2001–2004 (p < 0.01), whereas in the other age groups it decreased (significantly in the age group 15–24 years, p < 0.01).

When comparing persons 0–14 years of age to those 15 years of age and older, there was a significant increase (p < 0.01) in the percentage of cases represented by the younger age class, from 81.4% in 1991–1995 to 85.2% in 1996–2000 and 88.4% in 2001–2004.

The analysis by geographic area showed that, although the trend in incidence was similar for the three areas, there was a clear north-south gradient, with the highest incidence consistently found for northern Italy, followed by central and then southern Italy (Figure 1).

### Evaluation of other databases

In the period 2000–2005, 92,288 cases of varicella were reported to SPES, with the annual incidence ranging from 4,053 per 100,000 children (0–14 years) (2005) to 6,655 per 100,000 children (2004). In general, there was a trend of decrease in incidence from northern to southern Italy (Table 1).

**Table 1:** Varicella in Italy: SPES Survey, incidence/100,000 children 0–14 yrs, 2000–2005

Year	Northern Italy	Central Italy	Southern Italy	Italy
2000	6,034	5,234	4,852	5,340
2001	7,053	5,705	4,576	5,741
2002	5,605	6,168	5,031	5,459
2003	6,865	5,438	4,532	5,635
2004	7,297	6,400	6,120	6,655
2005	3,802	5,462	3,719	4,053

According to the National Hospital Discharge Database, in the period 2000–2003, there was an annual mean of 1,575 hospitalisations for varicella (1,521 hospitalisations and 54 day-hospital admissions). The mean duration of stay was 5.3 days. For zoster, in the same period, there was an annual mean of 5,250 hospitalisations (4,711 hospitalisations and 539 day-hospital admissions). The mean duration of stay was 8.3 days. Of the total hospital admissions for zoster, 62.1% were for persons greater than 65 years of age.

With regard to mortality, in the period considered (1991–2002), ISTAT reported 66 deaths for varicella (average of 5.5 deaths per year). In the same period, there were 416 deaths for zoster; 89% of these deaths were reported among persons older than 65 years of age.

### Seroprevalence study

**Figure 2:**
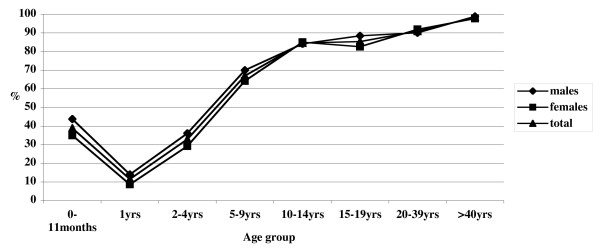
Varicella-zoster virus seroprevalence by age-group and gender in Italy, 2003–2004.

Overall, 2,367 samples were positive; 675 were negative, and 52 were equivocal. The seroprevalence followed a typical trend, with a decreased seroprevalence in the second year of life, compared to the first year (i.e., when children are initially passively protected by the mother). Beginning at 2 years of age, it progressively increased, reaching 32.9%, 67%, 84.6%, and 85.4%, respectively, in the age classes 2–4, 5–9, 10–14 and 15–19 years. In the age classes 20–39 and >40 years, the seroprevalence was 91% and 98.4%, respectively (Figure 2). The trend in seroprevalence was similar for males and females, with no statistically significant difference.

**Figure 3:**
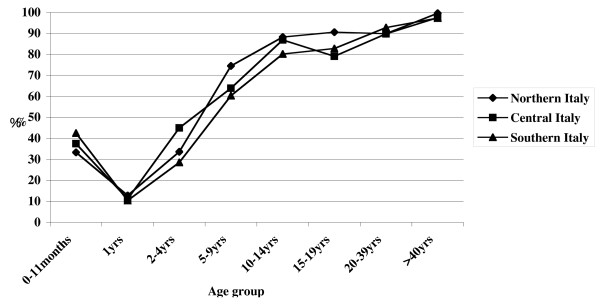
Varicella-zoster virus seroprevalence by age-group and geographical area in Italy, 2003–2004.

Analyzing data by geographical area (northern, central, and southern Italy), the trend in seroprevalence was basically uniform (Figure 3), though there were statistically significant differences when comparing the data for the following age classes:

- 5–9 years, northern vs. southern Italy, χ^2 ^= 8.70 (p = 0.003)

- 10–14 years, northern vs. southern Italy, χ^2 ^= 4.49 (p = 0.026)

- 15–19 years, northern vs. central Italy, χ^2 ^= 5.82 (p = 0.016)

- 15–19 years, northern vs. southern Italy, χ^2 ^= 4.68 (p = 0.043)

- ≥ 40 years, northern vs. southern Italy, χ^2 ^= 4.34 (p = 0.037)

However, multivariate analysis showed that the only variable associated with antibody titre is age group.

The GMT showed a trend of decrease in the first year of life and then a progressive increase in older age classes. This increase was statistically significant (p < 0.01) when comparing a given age class with the successive age class, except when comparing the 10–14-year age class to the 15–19-year age class, between which the GMT decreased, though not significantly. There were no significant differences when comparing males to females.

### Comparison of seroprevalence data from 1996 and 2004

**Figure 4:**
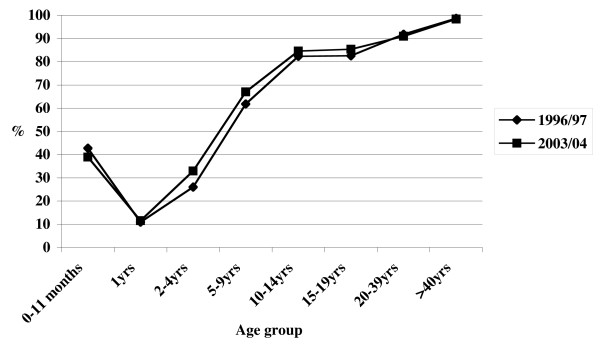
Comparison between varicella-zoster virus seroprevalence by age-group in Italy, 1996–1997 and 2003–2004.

The proportion of seropositive persons did not substantially differ from that reported in a study conducted using the same methods on samples collected in 1996–1997 [10]. No changes were detected in any of the age-groups, and the same trend was evident for both males and females (Figure 4).

## Discussion

Active and passive surveillance systems are extremely important for the evaluation of the epidemiological impact of an infectious disease. However, data collected through these systems have both strengths and weaknesses. For example, the national routine notification system adopted in Italy, being mandatory, is useful to describe the epidemiology of a specific infectious disease and allows the evaluation of historical temporal trends but is certainly affected by undernotification and underdiagnosis. Sentinel surveillance systems, such as SPES, being active surveillance systems, are certainly more precise, but their implementation is difficult and expensive. On the other hand, hospital discharge databases have poor sensitivity and a limited potential for clinical interpretation; however, they allow the evaluation of the more clinically serious infections. For all these reasons it is important, when possible, to evaluate different data sources to study the epidemiology of an infectious disease. The data from the statutory notification system for the years 1991–2004 confirm that varicella is a widespread infectious disease which mainly affects children. However, this system greatly suffers from underreporting. For example, in the period 2000–2004, the estimated incidence in the 0–14-year age class ranged from 1021.93 to 1368.31 per 100,000 population, whereas the incidence estimated based on data collected by SPES ranged from 5,340 to 6,655 per 100,000 population, which is consistent with the incidence of 6,200 per 100,000 population reported in a previous study and based on data provided by volunteer paediatricians [11]. These findings suggest that data collected by healthcare professionals on a voluntary basis may be more accurate than those collected by statutory notification systems. Based on the data provided by the statutory notification system and SPES, it can be estimated that in Italy there are approximately 500,000 cases of varicella each year [12] which correspond to a birth cohort.

The percentage of cases represented by persons ≥15 years of age progressively decreased, from 18% in the period 1991–1995 to 10.7% in the years 2001–2004. This seems to represent an inversion of the trend reported in a previous Italian study, in which the percentage of cases represented by persons ≥15 years of age progressively increased in the period 1961–1996 [10]; similar trends of increase have also been reported by other studies outside Italy [13]. Instead, the geographic differences in incidence (comparing northern, central, and southern Italy) were confirmed.

The seroprevalence study confirmed that in Italy VZV infection is predominantly a paediatric disease, and the results did not substantially differ from those of study conducted using the same methods on samples collected in 1996–1997 [10]. The trend in seroprevalence was similar for males and females and for geographical areas. Multivariate analysis showed that the only variable associated with antibody titre is age group. This is strongly suggestive that the epidemiology of VZV does not vary geographically in Italy. As a matter of fact, regional differences were particularly pronounced using the incidence data but much less so with the antibody prevalence results. These differences may be explained by the high degree of undernotification in southern Italy [12]. Also the higher seroprevalence registered in northern Italy may be related to the higher spreading of VZV in northern regions where a large part of the population lives in large urban and industrial settings.

The GMT showed a trend of progressive increase, indicating, as found in 1996–97, the existence of natural boosters deriving from the persistent circulation of the etiological agent. Nonetheless, it should be stressed that, for both genders, approximately 15% of adolescents and 9% of persons in the 20–39-year age class are susceptible.

In designing the sero-prevalence study, the representativeness of the sample was pursued following indications provided by the European Project for the Sero-epidemiological Surveillance of Vaccine-preventable diseases. Although the samples from very young subjects could have been collected from children affected by health problems, the inclusion and exclusion criteria adopted in this study should have allowed to exclude these children, thus avoiding an overestimation of seroprevalence.

This study shows that the sero-epidemiological profile in Italy is different from that in other European countries. In particular, in Italy, the percentage of persons who are susceptible is at least nearly twice as high as the percentages in the other countries participating in the European Seroepidemiology Network (ESEN) [14-17]. This confirms that there are important differences in the age of disease acquisition, and it is consistent with the finding that, among the ESEN countries, Italy has the lowest reproduction number (R_0_) and force of infection (calculated using seroepidemiological data from 1996–97) [18]. Moreover, in Italy, the high percentage of persons who are susceptible in the 20–39-year age class indicates that there is a concrete risk for VZV infection for pregnant women [19].

The results of this study can contribute to evaluating the options for varicella vaccination. As mentioned, before evaluating these options, it is necessary to have complete and updated epidemiological data, the acquisition of which entails not only performing seroepidemiological investigations but also creating or implementing passive or active surveillance systems for varicella and zoster. In Italy, reliable data for the 0–14-year age class are available from SPES, whereas the routine notification system suffers, as mentioned, from underreporting.

With regard to the actual vaccination options, mass vaccination in infancy (12–18 months of age), together with the vaccination of all susceptible persons by 13 years of age, would substantially decrease the spread of the disease and would protect more persons at high risk of complications [7]. However, it would be necessary to achieve high vaccination coverage rapidly, so as to avoid an increase in the mean age of acquisition of the infection and thus in the risk of complications.

There is a theoretical negative impact of mass vaccination in terms of increasing the incidence of zoster. In particular, given that exposure to VZV boosts natural immunity, the decreased circulation resulting from mass vaccination could potentially result in an increased number of cases of zoster [20] in first decades of a universal mass vaccination program.

Although in Italy there are no complete or updated epidemiological data on zoster, it has been estimated that in persons greater than 14 years of age there are approximately 200,000 cases of zoster, and 42,000 of post-herpetic neuralgia each year [21] and this risk of a further increase following mass vaccination needs to be carefully considered. However, data currently available do not support this hypothesis [22]. Moreover, the possibility of using a vaccine with a high antigen titer for preventing zoster in adults is being considered [23].

An alternative option would be that of vaccinating only susceptible adolescents and adults, which does not present problems related to vaccination coverage. Although this type of intervention would have a limited impact on the epidemiology of the infection, it would contribute to decreasing the incidence among persons at the greatest risk of complications. The analysis of the data from the National Hospital-Discharge Database showed that a small proportion of persons with varicella are hospitalised; in the period 2000–2003 (the years for which data were available), a mean of 1,575 hospitals admissions had varicella reported as the main diagnosis. However, considering that approximately one third of these cases occur among people older than 14 years, and that these cases could be prevented by the selective vaccination of adolescents and adults, this option merits consideration.

## Conclusion

Italy's 2005–2007 National Vaccination Plan [24] recommends vaccinating persons at high risk of complications, susceptible adolescents, healthcare workers, and the staff of day-care centres and schools with small children. Mass vaccination is only recommended in regions where a vaccination coverage of greater than 80% for MMR can be achieved. To date, national-level mass vaccination has not been performed, and the vaccination strategy varies greatly among Italy's 20 Regions: some Regions have already begun or plan to begin mass vaccination, whereas others have decided to vaccinate only susceptible adolescents, and still others have not decided on any type of intervention. The current strategy is quite different from that reported in the previous study, where the use of varicella vaccine was recommended only for individuals at risk, and the possibility of implementing vaccine campaigns targeting susceptible women and healthcare personnel was being considered.

A cluster sampling survey conducted in 2003 revealed a national MMR vaccination coverage of 77%, in children aged 13–24 months of age [25] and routine coverage data provided by Regions to the Italian Ministry of Health in 2004 showed an MMR vaccination coverage of 85.5%. It is therefore possible that in a few years, in all Italian Regions, there will exist the conditions necessary for implementing a mass vaccination campaign and that the large-scale availability of MMRV tetravalent vaccines in the near future will facilitate mass vaccination.

## Competing interests

The authors declare that they have no competing interests.

## Authors' contributions

GG, MCR, MLCDA and PC conceived of the study and participated in its design and coordination and helped to draft the manuscript. MG, ADD and AB acquired and analyzed data and have been involved in revising the manuscript critically for intellectual content. All authors read and approved the final manuscript

## Pre-publication history

The pre-publication history for this paper can be accessed here:


